# Traditional herbs against COVID-19: back to old weapons to combat the new pandemic

**DOI:** 10.1186/s40001-022-00818-5

**Published:** 2022-09-26

**Authors:** Hayder M. Al-kuraishy, Omnia Momtaz Al-Fakhrany, Engy Elekhnawy, Ali I. Al-Gareeb, Mohammed Alorabi, Michel De Waard, Sarah M. Albogami, Gaber El-Saber Batiha

**Affiliations:** 1 Department of Clinical Pharmacology and Medicine, College of Medicine, ALmustansiriyia University, Baghdad, Iraq; 2 Pharmaceutical Microbiology Department, Faculty of Pharmacy, Tanta University, Tanta, Egypt; 3 Department of Biotechnology, College of Sciences, Taif University, P.O.Box 11099, Taif, 21944 Saudi Arabia; 4 Smartox Biotechnology, 6 rue des Platanes, 38120 Saint-Egrève, France; 5 L’institut du Thorax, INSERM, CNRS, UNIV NANTES, 44007 Nantes, France; 6 Université de Nice Sophia-Antipolis, LabEx «Ion Channels, Science & Therapeutics», 06560 Valbonne, France; 7 Department of Biotechnology, College of Science, Taif University, P.O.Box 11099, Taif, 21944 Saudi Arabia; 8 Department of Pharmacology and Therapeutics, Faculty of Veterinary Medicine, Damanhour University, Damanhour, Egypt

**Keywords:** COVID-19, Phytochemicals, Traditional Chinese medicine, Bioactive metabolites, Nutraceuticals, Functional foods

## Abstract

**Background:**

Recently, the coronavirus (COVID-19) pandemic is a chief public health disaster caused by severe acute respiratory syndrome coronavirus 2 (SARS-CoV-2). There are no established effective preventive or therapeutic anti-COVID-19 drugs available except for some recently approved vaccines. Still, countless recent studies recommend various alternative and complementary approaches against COVID-19, which are medicinal herbs employed as traditional remedies to enhance immunity to struggle with viral infections. In addition, physicians worldwide are highly interested in vitamin and mineral supplements to help them combat COVID-19 either through protection or treatment. Dietary supplements specifically vitamin D, vitamin C, and zinc provide good prophylactic and therapeutic support to the presently available treatment regimens. In the present work, we have focused on plant-based remedies with promising anti-COVID-19 activities.

**Aim:**

To enable investigators and researchers to identify potential herbal compounds with anti-COVID activity to be used as promising therapies to combat this pandemic.

**Main body:**

This review highlights the recently published studies concerning natural traditional herbs, herbal bioactive metabolites, dietary supplements, and functional foods that could help prevent and/or treat COVID-19. Herein, we explored medicinal herbs as potential inhibitors of SARS-CoV-2 and discussed how these studies help form larger discussions of diet and disease. Moreover, by investigating the herbal bioactive components, we have outlined several medicinal herbs that can fight against COVID-19 by hindering SARS-CoV-2 replication and entry to its host cells, deterring the cytokine storm, and several other means. Finally, we have summarized various herbal products, functional foods, and dietary supplements with potent bioactive compounds which can inhibit and/or prevent COVID-19 disease progression.

**Conclusions:**

Based on the studies reviewed in this work, it was concluded with no doubt that phytochemical components present in various herbs could have a starring role in the deterrence and cure of coronavirus contagion.

## Introduction

**Fig. 1:**
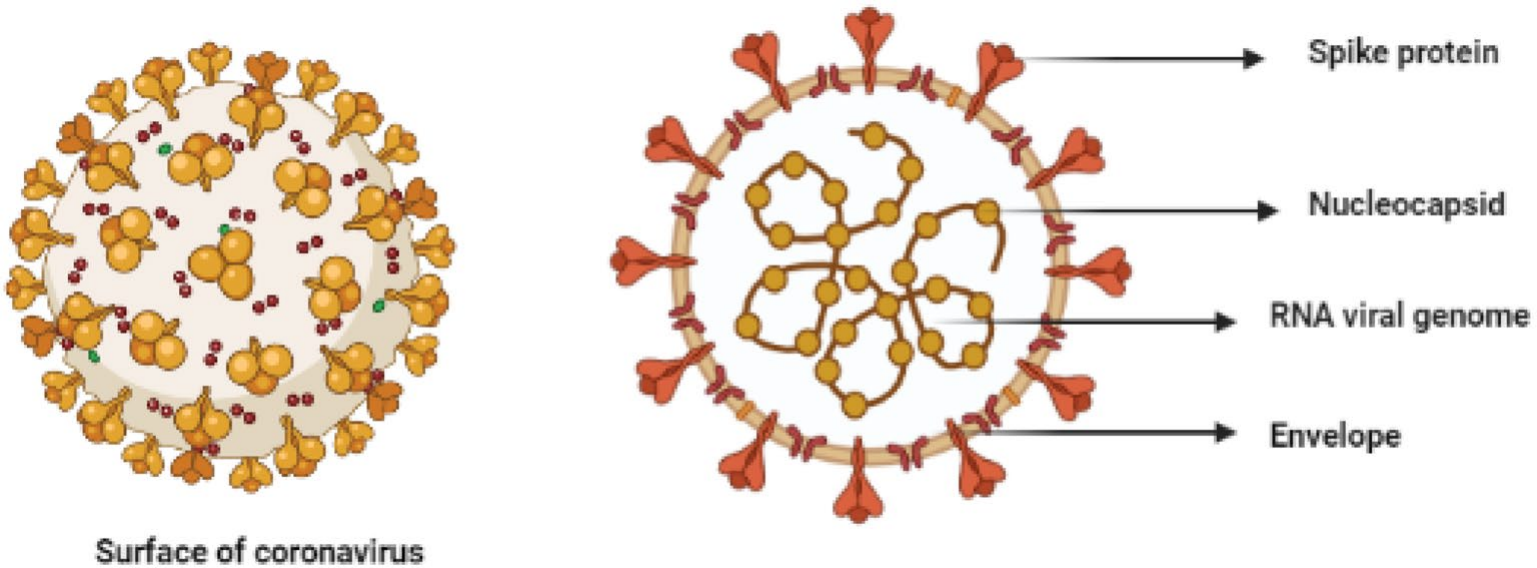
Structure of SARS-CoV-2 virus

The world is suffering the Coronavirus infection 2019 (COVID-19) which has been declared to be a global pandemic by the World Health Organization (WHO) in March [1, 2]. The coronavirus pandemic has been argued as the greatest crisis globally as this pandemic has emerged as the greatest worldwide health crisis since the influenza pandemic [3]. It caused more than 6.000.000 deaths all over the world [4]. The causative agent is severe acute respiratory syndrome coronavirus 2 (SARS-CoV-2) which is an enveloped beta-coronavirus that has non-segmented positive-sense RNA (Fig. 1). SARS-CoV-2 has about 30 kb of genetic information that is 70% identical to the SARS-CoV [5].

SARS-CoV-2 causes a disease in the respiratory tract of humans using the same receptor used by SARS-CoV; the angiotensin-converting enzyme 2. In severe cases, SARS-CoV-2 triggers an inflammatory immune response and the release of proinflammatory cytokines that result in many consequences. Cytokine storm, multi-organ dysfunction, and acute respiratory syndrome are some of such effects [6]. There is no definite, consolidated, and efficient remedy for COVID-19 infections. Several types of vaccines are now available to hinder the COVID-19 pandemic nonetheless their delivery is still a challenge, especially for developing countries [7]. Accordingly, the main concern for researchers is the innovation of reliable and effective antiviral therapeutics for SARS-CoV-2. Medicinal herbs may be an ideal gateway toward finding effective anti-COVID-19 therapeutics.

People worldwide, particularly from Asian counties including; Japan, India, and China, and also some African populations have employed herbs as remedies to alleviate symptoms of many diseases since the ancient age and are even up to date. This could be attributed mainly to the profound availability and relatively low cost of medicinal plants in these tribes [8]. Thus, it could be possible to develop novel drugs with possible anti-COVID-19 efficacy from herbs and their bioactive components [9]. Phytochemical metabolites, such as tannins, terpenoids, alkaloids, coumarins, flavonoids, and polyphenols, have shown efficacy against pathogenic microorganisms. This could be attributed to their ability to stop viral enzymatic and protein activities thus inhibiting viral entry and replication in the affected host cells [8]. Accordingly, quite a lot of reports have recommended the efficacy of herbal bioactive compounds in reducing and managing the risk of SARS-CoV-2 [8].

Evidence emphasizes that herbal medicine could be worthwhile in the management of COVID-19. Still, there is a shortage of information on the anti-COVID-19 agents derived from medicinal herbal extracts and their bioactivities [10]. The National Health Commission of China has permitted the usage of herb-based medicines in combination with Western medicine as an alternate therapy for COVID-19 and has authorized some commendations on the herbal therapy [10]. At present, herbal remedies having antiviral activities work as an auxiliary treatment to stop SARS-CoV-2 infections as the conventional treatment is quite not thriving [11].

Medication systems in various geographical zones use traditional medical herbs as the prime treatment for viral infections, including those triggered by the SARS-CoV. For instance, the leaf extracts of *Toona sinensis* suppress SARS-CoV replication [12]. In addition, licorice has been recommended as a promising for SARS-CoV treatment. Besides, natural products such as diterpenoids, triterpenoids, sesquiterpenoids, and curcumin have shown their ability to impede SARS-CoV infection [11]. Furthermore, dietary and herbal medicinal therapy may be efficient as an adjuvant against COVID-19. The bioactive ingredients in certain foods and herbs possess antioxidant, immunomodulatory, anti-inflammatory, and also antimicrobial activities. This could help in pre-and/or post-exposure prophylaxis by increasing the number and activity of cytokine suppressors, natural killer cells, lymphocytes, and macrophages. Consequently, the medicinal plant products could reduce the inflammation markers and respiratory disorders symptoms thus improving recovery [11].

Hence, this review aims to present the most important medical herbs and their bioactive compounds, and some dietary supplements and functional foods that possess potential antiviral activities regarding COVID-19.

### General overview on SARS-CoV-2 and the COVID-19 infection

SARS-CoV-2 is an enveloped RNA virus that belongs to the family Coronaviridae. This RNA virus has a single strand positive sense RNA genome [13]. Coronaviruses represent an assorted collection of viruses that infect several animals. Likewise, they cause respiratory illnesses with varying degrees of severity in human beings. In 2002 and 2012, correspondingly, two extremely pathogenic coronaviruses; the SARS-CoV (severe acute respiratory syndrome coronavirus) and the MERS-CoV (Middle East respiratory syndrome coronavirus), appeared in humans though having zoonotic origin. They triggered serious respiratory infections with high mortality, which made the emerging coronaviruses a novel public health concern in the twenty-first century [14]. By the end of 2019, a new coronavirus nominated as SARS-CoV-2 appeared in Wuhan, China. This novel SARS-CoV-2 caused an outbreak of uncommon virus-related pneumonia. SARS-CoV, the MERS-CoV, and the current pandemic triggered by SARS-CoV-2 have arisen as lethal CoVs able to cause severe respiratory tract contagions [15].

Genomic sequencing analyses have shown that SARS-CoV-2 fair resembles SARS-CoV using the same ACE2 to pass on a disease to its host. Accordingly, the possible targets for SARS-CoV-2 may be the expression sites of ACE2 [16]. On the other hand, the epidemiological dynamic parameters of SARS-CoV-2 are altered from the earlier human-CoV outbreaks having remarkable global spread [16]. Even though SARS-CoV-2 has more human-to-human transmission efficacy, its fatality rate (0.25–5%) is relatively less than that of SARS-CoV which is about 10%. Besides, SARS-CoV-2 has a basic reproduction number (R_0_) of 4.7–6.6 [16].

The infectious capability of SARS-CoV-2 is attributed to the greater affinity of its spike (S) protein for the ACE2 receptors, 10–20 times, more than SARS-CoV [16]. S-protein is a surface glycoprotein that aids to attach the virus to its host cell. Besides, SARS-CoV-2 has a furin-like cleavage site at the S1–S2 junction, lacking in other allies of its sister clade. The superior pathogenicity of SARS-CoV-2 could be attributed to this additional cleavage site, since it similarly occurs in the extremely contagious forms of influenza virus, however, missing in less infectious ones [17].

### Coronavirus origin and transmission

Bats are considered to be SARS-CoV-2’s natural host. SARS-CoV-2 is expected to have single or further more mammalian intermediate hosts for animal-to-human transmission. This could be ascribed to the ecological separation of bats from humans and the necessity of certain transmutations in the viral genome to evade the species barrier [18].

Human-to-human spread of SARS-CoV-2 is largely reported in hospitals, families, and communities [15]. The principal way of person-to-person transmission is droplet transmission. In addition, SARS-CoV-2 infection can spread via direct contact and fomite exposure [19]. In addition, contact with asymptomatic carriers is a possible route to transmit SARS-CoV-2. Owing to the exceptional pace with which SARS-CoV-2 spread, the airborne transmission also merits meticulous evaluation [20]. In addition, SARS-CoV-2 was detected in the fecal sample, even urine and saliva of corona-ill patients. Therefore, fecal–oral transmission could also be a possible route of viral transmission [17].

### Therapeutics/management

At first, early in the COVID-19 pandemic, our understanding of COVID-19 and its possible management was extremely limited. This created an urgency to alleviate the new viral infection using experimental therapies and even drug repurposing. Then, intense efforts of the researchers universally made substantial progress that has resulted in a better understanding of COVID-19 and its control. This has brought about the improvement of innovative therapeutic remedies and novel vaccines at an extraordinary speed [4].

### Herbal therapeutic approaches against COVID

The lack of precise effective therapeutics against SARS-CoV-2 has encouraged some investigators to shift toward plant-based therapeutic approaches. This is due to the fact that numerous drugs are either plant materials or derived from their bioactive herbal constituents. Consequently, there is a remarkable interest in the detection of prospective anti-COVID-19 herbal medicines, since plant-based therapeutics exhibited encouraging efficiency against various viruses via reinforcing immunity [1].

In the field of herbal medicine research, a single plant species may have great medical importance as it may contain a wide array of bioactive phytochemicals. These phytochemical components may act either alone or in a combination with other constituents to produce the wished pharmacological impacts. The beneficial effects of medicinal herbs come from their bioactive secondary metabolites comprising; alkaloids, steroids, triterpenes, and glycosides. Studying the pharmacologic impacts of plants is a challenge [21].

Currently, the main concern of medical research is the development of innovative antiviral agents. On top of displaying direct antiviral effects, herbal drugs having anti-inflammatory activity can have a major role in COVID-19 treatment. This could be attributed to the cytokine storm; elevation of the inflammatory mediators as erythrocyte sedimentation rate (ESR), interleukin (IL)-6, and C-reactive protein (CRP), which may cause severe disease with worse outcomes in COVID-19 patients [22].

### Traditional Chinese Medicine to treat COVID-19

Traditional Chinese Medicine (TCM) has been a crucial measure for the treatment and/or the prevention of some outbreaks. The TCM attained remarkable therapeutic consequences during the SARS epidemic in 2003. Throughout the COVID‐19 retrieval era, the TCM program has been involved in the COVID‐19 analysis and treatment guidelines and TCM experts were employed in the whole rescue procedure [23]. TCM has revealed promising effects in decreasing the rate of mild and/or severe cases, the overall mortality rate, and limiting the total disease duration. When herbal-based medicine is used in combination with modern biomedicine, it could exert direct antiviral, immunomodulatory, and anti-inflammatory effects, and also relieve both chronic obstructive pulmonary disease and hypoxemia [23].

Song et al. [24] reported the active perspective of *Sanctellaria baicalensis* extracts have baicalin that remains one of the main TCM herbal components along with hesperetin an active component in the tangerine peel. Both bioactive agents have been used for relieving the COVID-19 signs. An additional TCM medicine Xuebijing injection has remained broadly declared to reduce the possible hazards of community transmission of pneumonia in addition to the reduction of the time needed for patient ventilation in severe cases [24]. Moreover, Su et al. [20] suggested the efficacy of the traditional Chinese herb; *Exocarpium Citri grandis* in the treatment and hindrance of COVID-19.

An innovative attitude to TCM is a combination treatment that involves mixing the old traditional rehearses together to produce active formulations for various diseases. For example, the combination of the extract of *Radix Sophorae flavescentis* and the liquid fermented broth of *Ganoderma lucidum*, showed efficacy against the hepatitis B virus. In addition, glycyrrhizin, a bioactive constituent obtained from *Lycoris radiate,* exhibited a promising perspective as an anti-SARS-CoV therapeutic [25].

### Lianhua qingwen

Lianhua Qingwen (LHQW) is a traditional Chinese medication currently listed in the 2015 issue of the Chinese Pharmacopeia. LHQW is presented in various dosage forms including; capsule, granules, and decoction. It was formulated using 13 components [26]. It shows a broad range of antiviral utilities, chiefly owing to its immunomodulatory and inhibitory effect on virus reproduction, and its inhibitory impact on the pro-inflammatory cytokines. The therapeutic effects of LHQW on COVID-19 is based on its powerful binding capability with ACE2 and Mpro; which are therapeutic targets of SARS-CoV-2. Accordingly, it is confirmed to be advantageous for COVID-19 as a supplement and synergetic treatment approach [27].

### Qingfei touxie fuzheng recipe

Recent studies recommended consuming this recipe together with Western medicine for treating COVID-19. Combination therapy was more successful than Western medicine single-handedly. A possible approach could be the up-regulation of antiviral features and the down-regulation of pro-inflammatory mediators [28].

### Shufeng jiedu

Shufeng Jiedu capsule (SFJDC) is a traditional Chinese medicine often utilized to treat influenza. Now, it is recommended for the treatment of COVID-19. SFJDC stays promising as a combination therapy with arbidol for managing uncomplicated COVID-19 patients [12].

### Traditional medicine in India

Likewise, in India, traditional medicine is utilized for the management of COVID-19 together with the recent medicine and vaccinations. Traditional Indian medicine is considered one of the oldest elements that have a vital role in the global healthcare system [15]. These traditional rehearses involve siddha, unani, ayurveda, yoga, naturopathy, and homeopathy and they are efficiently practiced for curing various infections [15]. These traditional practices use animal yields, plants, and minerals for the management of several ailments. Roughly, 25.000 herb-based preparations and extracts have been employed in traditional medicine in South Asia.

In recent times, a decoction of maricha (*Piper nigrum*), lavanga (*Syzygium aromaticum*), and sunthi (*Zingiber officinaleRoscoe.*) have been suggested for both healthy and COVID-19 patients. The reason is that it supports the humoral and cell-mediated immunological reactions and depresses airway hypersensitivity. Likewise, the active component in the *Curcuma longa* Linn. curcumin, is documented to inhibit cytokine release, especially interleukin-1, interleukin-6, tumor necrosis factor-α, and the pro-inflammatory cytokines. It is advised to be taken with milk [22]. Blockage of cytokine release is regarded as one of the principal investigational elements for flu and several other contagions. Similarly, it has been applied to COVID-19, where cytokine storm has a key role in disease progression [22].

### Ayurvedic medicine to treat COVID-19

Ayurveda is the world’s ancient medicinal network that is assumed to be used in the management of various infections while having no adverse effects. Ayurveda is well-furnished with various treatment approaches for complicated deleterious ailments [29]. Ayurveda health care experts have been aware of various microorganisms and the infections triggered by them. The Ayurveda and the Siddha rehearse initially began in India and are quite widely used to cure numerous infections [25]. The identification, isolation, and characterization of bioactive phytochemicals in medical herbs might aid in the struggle with several infections. Thus, repurposing ancient medical plants possibly will provide a novel attitude for combating various viral contagions [25].

### Ayurvedic kadha

Ayurvedic medication and its extracts have been employed in the prevention and treatment of viral diseases. Kadha represents the earliest kind of medicine made by merging plant-based drugs and spices. It is an extract prepared from less juicy or dry constituents, such as herbs and spices from various Indian botanical drugs [30].

Making Kadha for oral consumption is an important Ayurvedic practice for augmenting the pharmacological effects of active components in botanic drugs. The Indian government recommended the usage of Kadha during the COVID-19 pandemic to improve the immune response and promote curing [30]. The Ayurvedic Kadha-based phytochemical constituents possess a potent binding affinity with several viral and host targets. A fact that proposes their antiviral activity through regulating virus replication in their host cells [30]. Recently, the Ministry of AYUSH (Ayurvedic, Yoga, Naturopathy, Unani, Siddha, and Homeopathy) in India commended drinking Kadha as a booster immunity and also for depressing the tenderness during the COVID-19 crisis [31].

### Guduchi ghan vati

Guduchi GhanVati is a traditional Indian remedy that is usually prescribed as an antioxidant and immunomodulatory therapeutic. Recently, its activity against the SARS-CoV-2 infections was also confirmed [32]. The common Ayurvedic preparation, Guduchi GhanVati is itemized in the Ayurvedic Pharmacopoeia of India. It is prepared from an aqueous extract of *Tinospora cordifolia*. *T. cordifolia* (Lour.) Merr., generally recognized as “Guduchi” or “Giloe” is a big climbing tree that belongs to the family *Menispermaceae*. It exists in the tropical areas of both China and India. It has been historically used in traditional medicine [1].

### *Tinospora cordifolia* and *Piper longum*

It is a herb present in India and China and belongs to the family of *Menispermaceae*. In the traditional Ayurvedic medication, this herb is utilized in the preparation of Guduchi Ghan Vati [33]. In addition, it could be administered in combination with *Piper longum* L. (family *Piperaceae*). *P. longum* is one more common medical herb employed in Ayurvedic medicines. *P. longum* is commonly known as “Indian Long Pepper” and “Pippali”. Pippali is recognized as a traditional Ayurvedic complementary constituent, which improves the absorption and bioavailability of other bioactive constituents. It also has notable antiviral activities [1].

### Plant secondary metabolites against COVID-19 infections

**Fig. 2:**
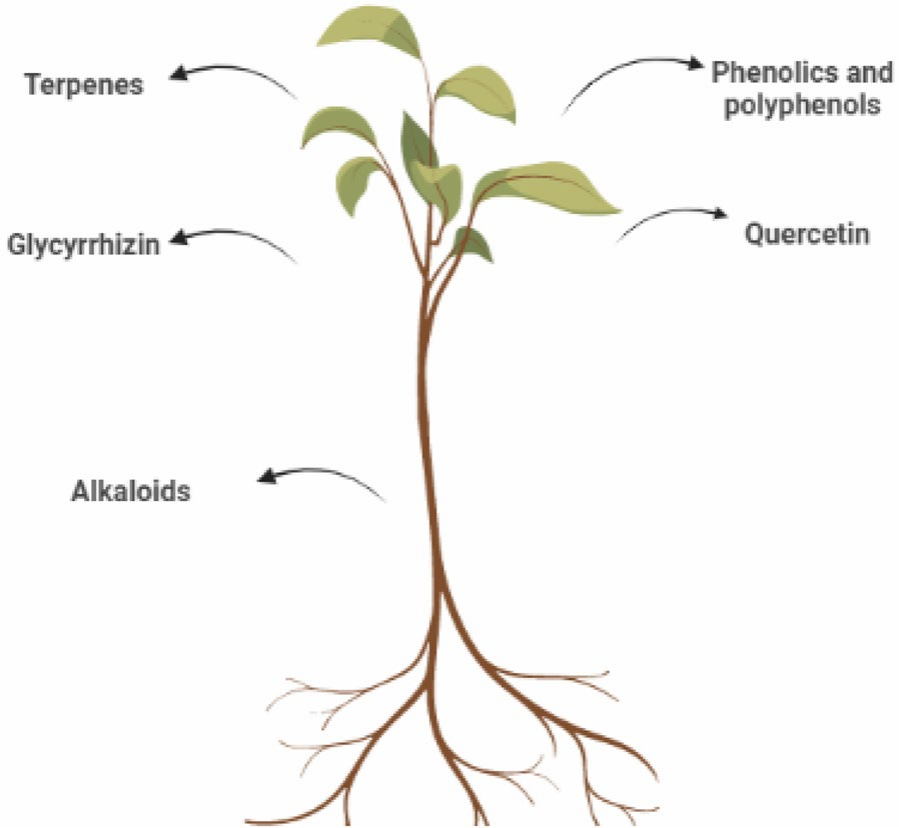
Most common bioactive metabolites in plants

Plant’s secondary metabolites (PSMs) are intermediate complexes formed as a result of stress exposure. PSMs can aid the host to interact and deal with various environmental stresses. They have strong antimicrobial, antifungal, and antiviral activities [34]. Recently, researchers revealed that PSMs may have antiviral properties in humans. Reviewing the recently available data concerning the utilization of plant active metabolites in the prevention and/or treatment of COVID-19 revealed the activity of several bioactive phytochemical components on various diseases. The possible mechanisms include crucial immunomodulatory activities, affecting COVID-19 biomarkers, or modulating or halting the SARS-CoV-2 itself. There are four main PSMs groups: terpenoids/terpenes, phenolics and polyphenols, glycosides, and alkaloids [35]. The main bioactive metabolites in plants are shown in Fig. 2. In this review, we discuss some of the newly published data as regards the application of plant bioactive metabolites in the prevention/management of COVID-19 infections.

### Terpenes (essential oils)

Terpenes are a large group of natural organic compounds present in plants. They are lipophilic compounds found in many plants' essential oils. Terpenes possess a strong odor that safeguards the plants from various pathogens [35]. Terpenes remain the principal secondary metabolites in more than 36,000 species. They could be used as anti-cancer, anti-inflammatory and antiviral, antioxidant, and also antibacterial [35]. Recently, terpenes have extended specific interest owing to their prominent antiviral activities. Terpenes can incorporate with the lipid bilayer of the virus disturbing its structure. Therefore, terpenoids are considered specific inhibitory compounds against viruses. Certain terpenes such as celandine-B, betulinic acid, and ursolic acid have displayed vigorous antiviral effects (IC_50_: 1–20 g/mL) [36]. Besides, the research discloses that terpenes have advanced binding affinities and strong inhibition with all coronavirus diversities and could be highly active against COVID-19. The outer spiky lipid layer of COVID-19 is important for its attachment to the host's cell membrane. Terpenes can destroy the lipid layer of COVID-19 and inhibit its binding characteristics [37].

Moreover, coronavirus consists of a single-stranded RNA. This RNA strand acts as an RNA messenger. Once it enters the host cells, it triggers the formation of two polyproteins that are further consisted of new replication and transmission complexes which regulate the RNA synthesis and structural proteins formation and also enhance protease enzyme activity. The protease enzyme plays a major role in the disintegration of the polyprotein [23]. The recent antiviral therapeutic strategies involve identifying inhibitors of protease enzymes from natural products. Among these, terpenes have a particular impact because of their diverse accessibility in plants and microorganisms and their little IC_50_ [32].

### Glycyrrhizin

Glycyrrhizin is a triterpene saponin that has several biologic activities and pharmacologic properties [23]. Recent studies have discussed the therapeutic potential of glycyrrhizin for the management of COVID-19. Glycyrrhizin has many activities as binding to angiotensin-converting enzyme II (ACE2), hindering the accumulation of intracellular reactive oxygen species (ROS), down regulating the proinflammatory cytokines, hindering thrombin, preventing the overproduction of airway exudates, and stimulating endogenous interferon [23]. Recently, it was reported that the combination of glycyrrhizin /and boswellic acids is valuable in the treatment of COVID-19 due to its multi-target mode of action. It is efficient in preventing mortality, shortening the recovery time, and improving the prognosis [38].

### Phenolics and polyphenols

Plant metabolites that have aromatic rings and one or more hydroxyl groups are called phenolics and polyphenols. In addition, they are known as polyhydroxy phenols due to the presence of multiple phenol structural units. They are considered valuable in the therapeutic field. Polyphenols include flavonoids, tannins, and rosemerinic acids. The hydroxyl group of polyphenols can interact with the positively charged amino groups of proteins thus resulting in their inhibition via destroying the 3-D structure of proteins. This unique intercalation with proteins caused the active inhibitory effects of polyphenols against various microbes and viruses [39]. Likewise, polyphenols can react with both the virus's protein and also DNA/RNA causing cell death. Consequently, numerous polyphenols are generally considered antiviral agents [36]. Concerning their antiviral, anti-inflammatory, and antimicrobial significance, polyphenols may also be a valuable source of research against COVID-19 infections [35].

### Quercetin

Quercetin originates from the Latin word “Quercetum” meaning the oak forest. Quercetin is chemically belonging to the flavones. It is the chief polyphenolic flavonoid, which could be obtained from several fruits and vegetables, such as berries, onions, apples, dill, lovage, capers, and cilantro. It could also be artificially received from supplementary tablets inclosing quercetin or some of its synthetic derivatives [40].

Quercetin displays major antiviral, pro-metabolic, and anti-inflammatory activities. Furthermore, researchers have discovered that quercetin supplements can support antioxidant, anti-inflammatory, anti-viral, and immunoprotective activities [41]. Recent studies have proposed the possible mechanisms by which quercetin can display its anti-COVID-19 properties. This include (a) disturbing SARS-CoV-2 S protein interaction with ACE2, which can inhibit viral entry into its host cells. (b) Preventing the SARS-CoV-2 replication. Nguyen et al. [42] have reported the inhibitory effects of quercetin against 3C-like protease (3CLpro), which is vital for SARS-CoV replication, with an IC_50_ of 73 μM. Besides, it was verified that quercetin interacts strongly with the SARS-CoV-2 Mpro, which is a protease assisting the virus RNA translation [42]. (c) Decreasing the cytokine storm owing to its anti-inflammatory activity [43].

### Alkaloids

Alkaloids are a wide group of secondary metabolites (≥ 12.000 compounds) that have at least one nitrogen atom in a negative oxidation state. Alkaloids can be advantageous in our search for anti-COVID-19 to manage this pandemic [44]. Alkaloids are mostly present in flowering plants, fungi, bacteria, and certain animal species. They are categorized according to their biosynthetic pathway as tropanes, quinolines, indoles, purines, isoquinolines, imidazoles, pyrrolidines, pyrrolizidines, pyridines, and other types. The pharmacological effects of these bioactive metabolites include antioxidant, antifungal, antimalarial, antibacterial, and antiviral activities [45].

Recent studies demonstrated the prospective effects of some alkaloids to be used either on their own or in combination with further medications for the management of the COVID-19 pandemic. The compounds reported with the greatest inspiring antiviral effects against SARS-CoV-2 that could be further explored by in vitro assays and clinical trials were: papaverine, caffeine, berberine, colchicine, crambescidin 786, cryptospirolepine, deoxynortryptoquivaline, cryptomisrine, 10-hydroxyusambarensine, emetine, ergotamine, camptothecin, lycorine, nigellone, norboldine, and quinine [45].

Colchicine is lipid soluble, a tricyclic alkaloid. It is currently used as an anti-inflammatory drug for gout, Adamantiades–Behçet’s disease, and other auto-inflammatory ailments, such as familial Mediterranean fever [46]. Besides, colchicine is explored against COVID-19 infection owing to its ability to interfere with inflammatory immune responses. In addition to its influence on neutrophil activity, colchicine has resulted in a decline in the production of superoxide free radicals, reduction in tumor necrosis factor, and indirect blocking of the NLRP3 inflammasome [47]. The NLRP3 inflammasome is a, which senses the threat and triggers local and/or systemic inflammatory responses through enhancing the pro-inflammatory cytokines, as the IL-1β [48]. Particularly, colchicine reduces the production of cytokines to manage the inflammatory activity and stop the cytokine storm [47].

In addition, colchicine is considered a microtubule disassembling agent as it hinders tubulin protein polymerization. This causes disastrous effects on microtubule polymerization. Coronaviruses depend on microtubules and the cytoskeleton to enter their host cells, and also to help in the transcription and replication of viral genome [46]. Theoretically, colchicine can inhibit the entry of coronavirus into the host cells, since the entry necessitates spike protein interaction with the cytoskeletal proteins, especially tubulin. It can further interfere with the coronavirus replication, because the microtubules are crucial to forming the double membrane vesicles in host cells, along with the assembly and the transfer of spike proteins into the virions, which are significant stages in the virus replication [1].

### Functional foods: complementary to combat COVID-19

The novel coronavirus global pandemic has caused high mortality with restricted availability of treatments. Due to a shortage of time, investigations, and adequate efficacy, most vaccines are unfledged or inaccessible to some societies. Nevertheless, numerous latest studies suggest several alternatives or complementary therapies for COVID-19, which include functional food. Functional food is also called nutraceuticals. They are food comprising bioactive compounds which have useful impacts on the customer’s health consumed as supplements or taken as whole food to give medicinal value. Functional food has raised popularity to prevent numerous diseases including COVID-19 [49]. Given the current COVID-19 pandemic, where there is no specific preventive or therapeutic agent accessible, a healthy immune system is considered one of the most important tools that should be well-thought-out [50]. Functional food with biological activities along with their nutritional properties are usually considered safer for consumption than synthetic drugs and have been receiving increasing interest recently as models for drug design [51]. Various functional foods may aid the body battle COVID-19 infection by hindering the production of pro-inflammatory mediators, reducing the expression of ACE2 receptors, and inhibiting essential enzymes in SARS-CoV-2 [52].

In general, minerals and vitamin supplements have been well-recognized to help the immune system fight viral infections. As the nutritional status is a key factor affecting the outcome of COVID-19 patients, physicians worldwide are largely interested in vitamin and mineral supplementation to help them combat COVID-19 either through protection or treatment [53].

### Important constituents of functional foods

#### Multivitamins

Functional foods contain several vital components, comprising vitamins. Vitamins are organic compounds necessary in our diet for the growth and development of the human body. Insufficiency of vitamins may obstruct the appropriate functioning of the body and lead to inadequate immunological reactions against various contagions [52].

#### Vitamin D

Vitamin D may decrease the hazards of infection and mortality in several ways. In recent times, it was reported that vitamin D has a starring role in decreasing the risk of the common cold via three probable strategies: cellular natural immunity, adaptive immunity, and physical barrier [54]. It augments cellular innate immunity by inducing antimicrobial peptides, such as human cathelicidin which shows direct antimicrobial activities against a wide spectrum of microorganisms, LL-37, and defensins [55]. Vitamin D is supposed to increase natural antibody production along with strengthening immunity through induction of monocyte differentiation and inhibition of lymphocyte proliferation [56]. Moreover, it can enhance the phagocytic activity of macrophages [55].

Furthermore, vitamin D may perhaps reduce the cytokine storm prompted by the innate immune system somewhat by reducing the expression of the pro-inflammatory cytokines, such as tumor necrosis factor α (TNF-α) and interferon γ (INF-γ). Thus the consumption of vitamin D diminishes the release of pro-inflammatory cytokines and increases the anti-inflammatory mediators’ expression by macrophages [55].

Recently, the DPP4/CD26 receptor has been reported to interact with the S1 domain of the COVID-19 spike protein. The expression of this receptor was found to be significantly decreased in vivo when the vitamin D deficiency was corrected. Optimization of vitamin D may diminish some of the critical downstream immunological sequelae accountable for worse clinical outcomes in COVID-19, such as sustained interferon-gamma response, and the persistent rise in interleukin 6. This is considered a negative prognostic indicator in acute pneumonia patients, as well as COVID-19 patients [57].

#### Vitamin C

There is a common belief that vitamin C enhances the immune system and can help in the treatment and/or prevention of respiratory tract infections [58]. Vitamin C is well-known for its antioxidant properties [59]. During infections, vitamin C levels become depleted and the patients generally need supplementation up to gram doses of vitamin C by intravenous administration, such as during severe sepsis [60].

Vitamin C is well-known to support the innate and also adaptive immune system by modifying the susceptibility to different viral infections, in addition to influencing inflammation. It can block the release of IL-6 from the endothelium induced by endothelin-1 (ET-1). Besides, vitamin C supplements can help in restoring the stress response [61]. Vitamin C may hinder the neutrophils from developing the neutrophil extracellular traps, which contributes to organ damage and death in COVID-19 patients [62]. Moreover, vitamin C may have favorable effects on thrombotic and/or thromboembolic diseases that are commonly present in COVID-19 patients [61].

Glycyrrhizic acid (GA) is the main phytonutrient present in licorice roots. It possesses antimicrobial, anti-inflammatory plus hepatoprotective activities. Recently, it has been reported for its binding ability with the ACE2 enzyme to inhibit the SARS-CoV-2 infection. Curcumin (CC) and its analogs are commonly utilized for their anti-cancer, anti-inflammatory, respiratory, and immune system benefits. In addition, they can suppress various cytokines. Recent studies showed that a combination of vitamin C, CC, and GA is expected to help in regulating the immunological response against COVID-19 infections and hindering the excessive inflammatory response to prevent the cytokine storm. Still, further in vitro/in vivo experiments are needed [63].

#### Minerals

Minerals are inorganic elements, needed in small quantities for various body tasks, including suitable functioning of the immune system. Some minerals are essential in greater amounts, such as magnesium, calcium, sodium, phosphorus, potassium, and chloride. Others are needed in little amounts, known as trace minerals, including; zinc (Zn), selenium (Se), copper (Cu), and iron (Fe). These serve crucial roles in many biological processes. Like vitamins, deficiency in such elements impedes health [64].

#### Zinc

Zinc is a trace mineral recommended to prevent viral replication and also hinder viral attachment to the nasopharyngeal mucosa. Recent studies proposed that zinc influences several respiratory pathogens, such as respiratory syncytial virus, rhinovirus, and the SARS-COV [65]. Zinc ions are linked with the normal differentiation, development, and functions of the immune cells. Hence, it is considered to be crucial for both the innate and the acquired antiviral immune responses [65]. Zinc deficiency may lead to reduced antibody production, decreased activity of the natural killer cells, low cytokine production, atrophy of the thymus, the chemotaxis and oxidative rupture of neutrophil granulocytes, and lymphopenia. Finally, Zinc may stabilize the cell membrane as a result contributing to the inhibition of virus entry to the host cell [32].

News studies are emerging to claim that zinc can have a starring role in COVID-19 control. This may perhaps be based on prior knowledge that some coronaviruses may cause the common cold. It is proposed that in coronavirus, zinc exerts its effects by interfering with viral polyprotein processing [66]. As the COVID-19 infection is accompanied by damage to the ciliated epithelium and ciliary dyskinesia, zinc supplements may help to fight COVID-19. This is could be explained based on the reports that showed that the physiological concentration of zinc increases the ciliary beat frequency. In addition, zinc will help enhance the immune system to combat any other bacterial and fungal co-infections that contribute to mortality in COVID-19 patients [67]. Notably, there is a functional relationship between zinc and ROS (reactive oxygen species) production in platelets [68]. This indicates that zinc could reduce thrombus formation, a critical complication in COVID-19 ill people [66]**.**

#### Nutraceuticals supplements

Nutraceuticals are products consumed to enhance good health, improve life expectancy, slow down aging, guard against chronic illnesses, and/or help the body normal function [69]. Nutraceuticals come from herbs, particular diets, dietary supplements, and processed food. Recent investigations have pointed out the efficacious use of nutraceuticals for the treatment of various ailments including; diabetes, atherosclerosis, cancerous, and neurologic disorders [70]. The most commonly used dietary supplements and nutraceuticals are summarized below.

#### Ginseng

Ginseng enhances the B-lymphocyte proliferation and promotes the production of inflammatory mediators, including INF-γ and interleukins, that affect the immune system activation and regulation. Besides, Ginseng has anti-inflammatory activities and hinders viral replication [71]. Yet, the therapeutic immunomodulatory effects of ginseng are not distinctive. Many researchers have faith in ginseng to help treat infections in the upper respiratory tract and diminish the common cold and flu, yet there is no well-founded data to support this belief in the COVID pandemic [72].

#### Omega-3 fatty acid

Omega-3 is a kind of phospholipid present in the cell membrane, and hence it is significant for the pulmonary, cardiovascular, endocrine, and immune systems to do their functions appropriately [73]. It affects the immune system via modulating the activity of neutrophils, macrophages, B cells, T cells, natural killer cells, and other immune system cells. Regarding COVID-19, greater doses of omega-3 may decrease the severity of the illness. However, researchers recommend its use in the COVID-19 pandemic due to its confirmed and established capability as an anti-inflammatory and immune-stimulating agent [74].

## Conclusions and future perspectives

Various nations are suffering multiple waves of COVID-19 with growing stress on the health care system. Investigating plant-based therapeutic approaches including traditional medicines, bioactive metabolites, and functional foods to combat the SARS-COV-2 could provide massive achievements in our battle against the COVID pandemic. The significant therapeutic contributions of herbs and/or their active metabolites in the past made several recent research suggest their use as valuable therapies for COVID-19. They may be used alone or as complementary or alternative medicines to be able to combat COVID-19 infections. Our review indicates that many promising bioactive metabolites, plant-based herbal preparations, nutraceutical products, and functional foods showed encouraging anti-COVID effects, and they are in various stages of clinical trials to allow their use in the treatment of COVID-19. These herbal remedies may not prevent viral infection but can enhance the patient's welfare by supporting immune system preservation. Still, the evaluation of the effectiveness and safety of these phytochemicals and herbal preparations is crucial to exploit their therapeutic properties for the management of COVID-19 patients. As a final point, COVID-19 is challenging human beings all over the world. Dealing with this pandemic necessitates efforts of each individual and global cooperation of researchers, experts, and authorities. Besides traditional herbs, numerous functional foods, healthy lifestyle choices, and dietary supplements can considerably lessen the financial strain of COVID-19 suffering patients and the death rates across the world during this pandemic.

## Recommendations

The data reviewed recommends that herbs and their bioactive compounds should be considered either alone or in combinations with anti-COVID 19 potentials in future clinical studies.

## Data Availability

All data are available in the manuscript.
